# Natural Product Target Network Reveals Potential for Cancer Combination Therapies

**DOI:** 10.3389/fphar.2019.00557

**Published:** 2019-05-31

**Authors:** Steven R. Chamberlin, Aurora Blucher, Guanming Wu, Lynne Shinto, Gabrielle Choonoo, Molly Kulesz-Martin, Shannon McWeeney

**Affiliations:** ^1^Division of Bioinformatics and Computational Biology, Department of Medical Informatics and Clinical Epidemiology, Portland, OR, United States; ^2^OHSU Knight Cancer Institute, Portland, OR, United States; ^3^Oregon Clinical and Translational Research Institute, Portland, OR, United States; ^4^Department of Neurology, Oregon Health and Science University, Portland, OR, United States; ^5^Departments of Dermatology and Cell, Developmental and Cancer Biology, Oregon Health and Sciences University, Portland, OR, United States

**Keywords:** natural product, antineoplastic drug, cancer, synergy, therapeutic targets

## Abstract

A body of research demonstrates examples of *in vitro* and *in vivo* synergy between natural products and anti-neoplastic drugs for some cancers. However, the underlying biological mechanisms are still elusive. To better understand biological entities targeted by natural products and therefore provide rational evidence for future novel combination therapies for cancer treatment, we assess the targetable space of natural products using public domain compound-target information. When considering pathways from the Reactome database targeted by natural products, we found an increase in coverage of 61% (725 pathways), relative to pathways covered by FDA approved cancer drugs collected in the Cancer Targetome, a resource for evidence-based drug-target interactions. Not only is the coverage of pathways targeted by compounds increased when we include natural products, but coverage of targets within those pathways is also increased. Furthermore, we examined the distribution of cancer driver genes across pathways to assess relevance of natural products to critical cancer therapeutic space. We found 24 pathways enriched for cancer drivers that had no available cancer drug interactions at a potentially clinically relevant binding affinity threshold of < 100nM that had at least one natural product interaction at that same binding threshold. Assessment of network context highlighted the fact that natural products show target family groupings both distinct from and in common with cancer drugs, strengthening the complementary potential for natural products in the cancer therapeutic space. In conclusion, our study provides a foundation for developing novel cancer treatment with the combination of drugs and natural products.

## Introduction

While treatment for cancer has seen great strides in recent decades, we still face many open challenges in cancer therapy. In particular, adaptive resistance to cancer therapies has necessitated a move into rational combination therapies if we are to achieve a sustained therapeutic benefit for patients. An active area of interest is the inclusion of natural products in combination therapies for cancer treatment. In this work, our core premise is that the target space associated with natural products (NPs) will increase the number of potentially therapeutically accessible targets and lead to novel combination therapies for cancer treatment. To investigate this premise, we will not only quantify these new therapeutic targets and associated molecular pathways, but also assess the functional qualities and complementarity with FDA-approved cancer drugs of this space by using a variety of network methods.

Currently, the National Cancer Institute lists eight categories of cancer treatments: surgery, radiation, chemotherapy, immunotherapy, targeted therapy, stem cell transplant, and precision medicine^1^. Historically, surgery, radiation, and chemotherapy were the primary forms of treatment. In the late 1990s the FDA started approving targeted therapies for cancer, i.e., therapies directed toward unique molecular characteristics that drive oncogenesis. The first such therapy, imatinib, has shown an 80% decrease in 5-year mortality with chronic myeloid leukemia patients (Druker, 2009). While some of the early targeted therapies have resulted in dramatic clinical responses, drug resistance often develops after an initial positive response. This adaptation to treatment is known as acquired drug resistance, as opposed to intrinsic resistance, which exists prior to any cancer therapy (Holohan et al., 2013).

Acquired drug resistance is seen with both cytotoxic chemotherapies and targeted therapies, although mechanisms differ. Knowledge of the molecular mechanisms of resistance can inform therapeutic strategies. In cancer, these mechanisms can include compensatory and redundant molecular signaling, target mutations acquired during treatment, increased expression of the targeted proteins, inactivation of pro-apoptotic pathways, inhibition of DNA repair mechanisms, epithelial-mesenchymal transition, activation of pro-survival signaling, and upregulation of tumor cell efflux transporters (Holohan et al., 2013; Housman et al., 2014). For drug resistance caused by mutations in drug targets or redundant cell pathways, “rational combinatorial targeted therapy” is a possible solution (Al-Lazikani et al., 2012). This “rational” approach is done within the framework of network pharmacology, which brings together systems biology, network analysis, redundancy, and consideration of all drug target effects, beyond therapeutic intention, for designing therapies (Hopkins, 2008). Knowledge of molecular signaling pathways can be used to design multi-target strategies to block redundant pathways or newly mutated targets. Simultaneous targeting of multiple cancer hallmarks is another approach (Hopkins, 2008). Along with reduction in drug resistance, this approach can also lead to decreased adverse effects and increased efficacy (Hanahan and Weinberg, 2011). For these combinations, multiple therapeutic agents can be used, but these methods can also take advantage of poly-pharmacological characteristics of each single agent (Hu et al., 2014). Drugs can also work together through pharmacokinetic mechanisms, coalistic mechanisms (Jia et al., 2009), and through independent actions when used in combination (Palmer and Sorger, 2017). A interaction is coalisitic when two compounds interact in a biological context to form a new third compound.

Natural products can be broadly defined as any compound derived from a living source (animal, plant, microbial, fungi). This absolute definition would include “natural” cosmetics, “natural” foods, wood, silk, bioplastics and even coal. A more specific definition was based on the following from the National Center for Complementary and Integrative Health (NCCIH) (Milshteyn et al., 2014):

“Natural products include a large and diverse group of substances from a variety of sources. They are produced by marine organisms, bacteria, fungi, and plants. The term encompasses complex extracts from these producers, but also the isolated compounds derived from those extracts. It also includes vitamins, minerals and probiotics.”

We have further refined this definition with the following inclusion and exclusion criteria. Only NPs from plants and fungi were included, and only isolated compounds. In addition, only plants and fungi that have a history of traditional medicinal use were included. These compounds already have some historic use as therapeutic agents, and some are already classified as safe for human consumption by the FDA's Dietary Supplement Health and Education Act of 1994.

Natural product compounds show greater structural diversity, bioactivity and complexity than compounds in synthetic drug libraries, have the ability to inhibit some targets considered “undruggable,” such as protein-protein interactions, and inherently target biologically relevant space since they are mostly secondary metabolites, or signaling molecules (Harvey et al., 2015). There is also limited overlap between the molecular space targeted by natural products and targeted by synthetic drug libraries (Harvey et al., 2015). These characteristics not only indicate the potential for new targets for therapy, but also can help reduce the cost of the development of new treatments, since these molecules already exist in nature, and offer additional options for combination therapies. Natural products, or natural product derivatives, are the source of 33% of cancer drugs developed between 1981 and 2014 (Newman and Cragg, 2016). While the use of natural products for cancer treatment by patients is sometimes considered “complementary medicine,” there is also a body of literature demonstrating *in vitro* and *in vivo* natural product synergies with cancer drugs (Cote et al., 2015; Cheng et al., 2016), overcoming drug resistance with the addition of natural products (Pearson et al., 2017), and paradoxical synergy in cancer cells while demonstrating antagonism in healthy tissue (Cote et al., 2015). Therefore, the exploration of the natural product target space could offer the potential to improve existing drug therapy outcomes and reduce side effects. Computational methods, including approaches in graph theory (Sun et al., 2015) and assessment of differential gene expression (Liu and Zhao, 2016), have been successfully developed to predict synergy between compounds *in silico*. Predicting these effective drug combinations often requires up-to-date, comprehensive knowledge of the target space associated with a set of compounds.

**Figure 1:**
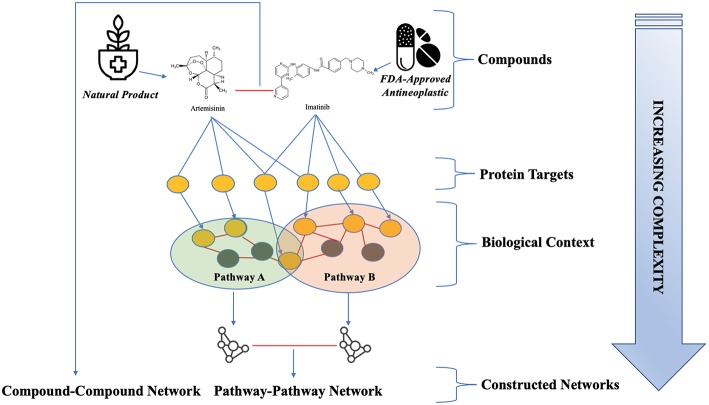
Framework for natural product target network evaluation. The targets associated with both NPs and anti-neoplastic drugs were evaluated in different contexts of increasing complexity. Complementary and distinct coverage of protein targets and pathways by the two compound classes were assessed. Target importance and relationships were evaluated in biological contexts, which include protein-protein interaction networks and molecular pathways. Pathway relationships and shared target space were assessed through the construction of a pathway-pathway network and a compound-compound network. Red lines indicate the existence of an edge between nodes in these networks. Two compounds have an edge if they share at least one target, and two pathways have an edge if they share at least one protein (Mahira and Umehara, 2018; Mykhal, 2004; Hinemash6, 2010).

However, natural product-target information in the public domain has several high need areas to address if we are to harness natural products in computational methods for predicting and prioritizing compounds for high throughput screens. There is a need not only to compile a comprehensive set of natural product targets from public sources, but also to characterize these compound-target relationships with respect to their supporting evidence, within a variety of relevant contexts (Figure 1). Such an evidence- characterization was recently completed for all FDA-approved antineoplastic drugs from multiple public resources by Blucher, et al. in the Cancer Targetome (CT) (Blucher et al., 2017). The methods and evidence framework from that project were applied here to develop and characterize a natural product target network (natural product compounds linked to associated targets). By comparing our results with natural products to the results of the Cancer Targetome, we provide a quantitative assessment of the potential of natural products, when added to FDA-approved cancer drug space, to increase novel cancer therapies. This characterization of the natural product network includes assessment of target and pathway coverage, compound promiscuity, established cancer driver genes, and the potential to inhibit molecular compensatory signaling mechanisms. This characterization of NP target space serves to not only identify potential new targets, but also evaluate the importance of these targets in a molecular context for therapeutic targeting in cancer treatment. In particular, we evaluate the dual space of natural products and FDA- approved cancer drugs from the Cancer Targetome for potential synergy in drug combination therapy. To assess this potential, we propose using a variety of network methods to evaluate natural products and their targets in the context of biological networks. First, by mapping natural product targets of interest into large protein-protein interaction networks, we can characterize their importance to the network using measures of “centrality” as well as their proximity to known cancer driver genes. Second, we create a network where entities are biological pathways, which allows us to assess potential pathway cross-talk. This is critical for efforts to prioritize combinations of natural products and FDA-approved drugs to interfere with pathway-based mechanisms of drug resistance. Finally, we create a network that contains both the natural product compounds and FDA-approved cancer drugs as entities, with connections between entities indicating shared protein targets. This network allows us to assess targets with respect to class of compound and can potentially be harnessed in rational combinations, such as combining compounds that target different families of targets for a complementary treatment strategy.

## Methods

### Data Collection

Seven public databases were used to construct the natural product target network used in this project, as sources of both compounds and targets (Table 1). Two of the seven databases were the source of natural product chemical compounds, TarNet (2016) (Hu et al., 2016), and the Traditional Chinese Medicine Integrated Database for herb molecular mechanism analysis (TCMID, version 2.01) (Xue et al., 2013). These databases were chosen because they contain only compounds from plants used from medicinal traditions.

**Table 1:** Public resources for natural product ligands and associated target interactions.

**Database**	**Ligands?**	**Target interactions?**
TarNet	Yes	Yes
Traditional Chinese Medicine Integrated Database (TCMID)	Yes	Yes
DrugBank	No	Yes
Therapeutic targets database	No	Yes
International Union of Basic and Clinical Pharmacology (IUPHAR)	No	Yes
BindingDB	No	Yes
Universal Natural Products Database (UNPD)	No	Yes

Four of the target source databases used were not specific to natural product compounds and were also used in the creation of the Cancer Targetome. These included DrugBank, version 5.0.7 (Law et al., 2014); Therapeutic Targets Database, version 4.3.02 (Qin et al., 2014); the International Union of Basic and Clinical Pharmacology (IUPHAR)/British Pharmacological Society (BPS) database, version 2017.4 (Harding et al., 2017); and BindingDB (7/1/2017). Additional human target interactions were included from three natural product databases. These include TarNet, TCMID and the Universal Natural Products Database (Gu et al., 2013). For full details of data analyzed in this article see Data Sheet 1.

### Base Natural Product Compounds

The TarNet database, the first of the two sources of NP compounds, contains information about 12,187 compounds derived from 894 medicinal plants. These plants are used in four traditions of botanical medicine: Chinese, Japanese, European and American (Hu et al., 2016). This database also contains 10,783 targets associated with the plant compounds through direct regulation or indirect effect. Both the plant-compound and compound-biotarget relationships were derived by text-mining and manual curation. A random sample of 91 literature references, used by TarNet to support the compound-biotarget relationships, was manually checked for the presence of binding affinity data in the source.

The second source, TCMID, comprises plants, associated compounds, and bio-targets curated from Traditional Chinese Medicine (TCM). The goal of this database is to translate the common factors between modern western medicine and TCM. The TCMID database contains 8,159 plants, 43,413 associated compounds, and 17,521 bio-targets compiled through a combination of other databases and text mining (Hu et al., 2016; Traditional Chinese Medicine Integrated Database, 2018).

The compounds from these two databases were combined and redundancies were removed. This was done through the use of the multiple keys associated with each compound in each database. TarNet compounds were classified by chemical name, Chemical Abstracts Service (CAS) number, simplified molecular-input line-entry system (SMILES), and International Union of Pure and Applied Chemistry's International Chemical Identifier Key (INCHIKEY). TCMID compounds were classified by PubChem Compound ID, SMILES and chemical name. The Chemical Translation Service (CTS) from the University of California at Davis was used to help resolve some of the missing key data in TarNet (Wohlgemuth et al., 2010). The two compound lists were then combined and scrubbed for possible drugs that are not of NP origin using lists from DrugBank (Law et al., 2014) and the FDA. The five keys found in the source databases: chemical name, SMILES, INCHIKEY, Pubchem Compound ID, and CAS number, were then used to retrieve target information from a variety of sources as detailed below.

### Biological Target Retrieval

Molecular target interaction information for the NP compounds was retrieved from seven publicly available data sources. Four widely used data sources were chosen based on the rationale used by Blucher et al. for the Cancer Targetome (Blucher et al., 2017). These sources include DrugBank (Law et al., 2014), Therapeutic Targets Database (Qin et al., 2014), IUPHAR/BPS Guide to Pharmacology (Harding et al., 2017), and BindingDB (Gilson et al., 2016). These four sources contain substantial information about NPs but are not limited to this class of compounds. Three additional sources of target interaction data that are limited only to NPs were also used. These include TarNet (Hu et al., 2016), TCMID (Xue et al., 2013), and the UNPD (Gu et al., 2013). All of the interaction data used for this project were based on experimental data from the literature, no computationally predicted interactions were used.

These data were retrieved by systematically merging each of the five NP keys, from the base NP data, individually to the seven target data sources. The data retrieved by the individual key were then associated back to the unique five key combination of values from which they were derived and data redundancies were resolved to create the final NP target network database.

The Evidence Level framework developed by Blucher et al. (2017) was then applied to these data as follows. Each NP-target interaction could have more than one piece of supporting evidence. Each piece of evidence was assigned one of three levels. Evidence Level I had an entry in only one of the databases for the interaction, without a supporting literature reference or experimental binding value. Evidence Level II had a supporting literature reference in the database but without binding affinity value, and Evidence Level III had both an experimental binding value and a literature reference. For NP-target interactions with multiple evidence entries from different databases, the maximum Evidence Level was assigned. For a single target, the maximum Evidence Level from all associated NP-target interactions was assigned. Analyses for this project considers all levels of evidence when appropriate and also focuses on interactions only with evidence of strong binding affinity, < 100 nM (IC50, EC50, Ki, or KD). Less stringent levels can still be considered biologically relevant, but below 100 nM is considered significant for drug binding (Paolini et al., 2006; Wang et al., 2016).

### Target, Pathway, Tumor Type, and Cancer Driver Coverage

Analyses compared the two target networks above, i.e., the NP target network and the Cancer Targetome (CT). First, the sets of targets associated with the NP and CT drug networks were mapped to the two associated sets of molecular pathways. The Reactome Pathway Knowledgebase (Fabregat et al., 2017), which leverages biological entity reactions, was the source of the pathway information. A total of 1,944 hierarchically structured pathways was used from this database for the first pathway mapping. For the second pathway mapping, a set of pan-cancer aberrant pathways was derived from the full set of Reactome pathways by performing an over-representation analysis with likely cancer driver genes. The genes cataloged for the Cancer Genome Interpreter (Tamborero et al., 2017) were used for this analysis. These genes have either experimental, clinical or *in silico* evidence showing that their mutations can drive tumorigenesis. There are 837 genes cataloged, representing 193 different tumor types. A hypergeometric test identified pathways enriched with these driver genes. The Benjamini-Yekutieli method was used to control the false discovery rate for multiple testing with dependencies (Benjamini and Yekutieli, 2001). If a pathway contained at least one molecular target with evidence of an interaction with either a NP or a CT drug, then the entire pathway was considered targeted by the respective compound category. Pathways were then classified as either targeted by NP only, CT drugs only, both NP and CT drugs, or neither. This same mapping classification was also applied at the protein target level, as opposed to the pathway level, to all of the targets associated with both target networks, and to targets only associated with the pan-cancer aberrant pathways. Finally, tumor types were identified that were associated with cancer driver genes targeted only by NPs. Only high-affinity interactions (IC50, EC50, Ki, or KD < 100 nM) were considered for this analysis.

The multi-targeting aspect of these two compound classes (NP and CT drug) is considered the basis for their poly-pharmacological effects (Hu et al., 2014). These effects can be undesirable, such as adverse events, or they could be the mechanism of the therapeutic effect. For this reason, it is important to map and compare the average interactions (targets, pathways, tumor types, and cancer drivers) per compound between the two target networks and to understand how NPs might differ from the CT drugs. For most analyses in this project we considered only < 100 nM target interactions, but for this analysis we considered two binding affinity thresholds: < 1,000 and < 100 nM. Distributions of interactions per compound were then compared between NPs and CT drugs for the four categories mentioned. A two sample Kolmogorov-Smirnov test was used to compare the two distributions, NP and CT drugs.

### Molecular Interaction Network Topology

For molecular interaction analysis, the NP targets, CT drug targets and cancer driver gene products were projected onto biological networks, and the topological features of these targets were evaluated and compared. Target-oriented topology research often uses a large non-specific protein-protein interaction network (PPI) for the biological context, but it has also been suggested that the base network should be either be more specific to the tissue or disease of interest, or have greater biological relevance (Peng and Schork, 2014). For this reason, two specific interaction networks were used for this evaluation. For the first biological network, the protein functional interactions from the Reactome Functional Interaction Network were used. This network integrates uncurated relationships from sources such as PPI databases and others with curated interaction information derived from pathway data in Reactome and other databases (Wu et al., 2010). These functional interactions have a higher likelihood of being functional in a biological context than an interaction from an uncurated PPI database. The second network is a PPI constructed by Wang (2014) from the integration of four manually curated human cancer signaling networks with protein interactions from BioGRID (Awan et al., 2007; Cui et al., 2007; Li et al., 2012; Newman et al., 2013;Chatr-Aryamontri et al., 2015).

Network measures that were considered include degree centrality, betweenness centrality, eigenvector centrality, and average shortest distance to cancer driver genes in the network. Degree centrality is the number of connections a node in a network has to neighboring nodes. In a biological network this could be the number of different interaction partners a single protein might have. Betweenness centrality is the number of shortest paths in a network that pass through a specific node. In a biological network this measure can capture the node's ability to control communication (Peng and Schork, 2014). Eigenvector centrality is a measure of how connected the nodes are that are connected to the node of interest. This is a measure of importance of the node's neighbors in the biological network. The average shortest distance is the path between two nodes that contains the minimum number of nodes, or steps. In a biological network this can be related to the number of reaction steps between two proteins (Yildirim et al., 2007; Li et al., 2012). Proteins that are closer could have a higher likelihood of impacting each other, if used as a therapeutic target.

To compare the topology measures, the nodes in the network were classified in four ways: targeted only by NPs, targeted only by CT drugs, targeted by both, and not targeted by either. These were compared to each other for similarities and differences to evaluate any additional therapeutic potential achieved with the addition of NPs. Only targets with strong binding affinities, < 100 nM, were considered.

### Compensatory Pathways

Chen et al. (2016), constructed a pathway-pathway interaction network to evaluate drug pair synergy and as a possible model for the inhibition of pathway crosstalk. In this network, a node represents an entire molecular pathway and an edge represents an interaction between two pathways. Three types of interactions were used to construct three networks. The three edges were defined by manually curated interactions from the KEGG database (Kanehisa and Goto, 2000), protein-protein interactions, and shared genes. Two sets of pathways were defined by associating the two drugs via the projection of the associated drug target networks onto the pathways. Chen et al. found that drug synergy was correlated with the average shortest distance between the two sets of pathways in this network, most strongly in the network with edges representing shared genes.

This structure was chosen as a good method to evaluate the NP and CT drug target network's crosstalk inhibition potential, since it is possible to measure global patterns in this framework and assess the two target sets in their entirety. Pathways in this network were then classified as being associated with NPs only, CT drugs only, both or none. A compound was associated with a pathway only if there was at least one target with a strong binding affinity, < 100 nM, in a pathway. Average shortest path distance between NP associated pathways and CT associated pathways was assessed to estimate the possible synergistic potential between NPs and CT drugs, and also to estimate increased cross talk inhibition by considering NPs. Reactome entity level pathways (non-hierarchical) were chosen to construct the network, of which there are 290. In addition, the previously described cancer driver genes were used to do an over-representation analysis on this set of 290 pathways, identifying possible aberrant cancer pathways in this network. Orientation to these cancer pathways in this network was then assessed for the pathways associated with NPs only, CT drugs, and both NPs and CT drugs.

### Compound-Compound Network

In this network, a node represents a compound, either NP or CT drug, and an edge exists if they share at least one target, both having a binding value of < 100 nM. The edges were also weighted based on the number of targets shared. This network analysis has also been used for FDA approved drugs (Yildirim et al., 2007). Network communities were identified and assessed for enrichment in either NPs or CT drugs to see if they are clustering around the same targets, or separate sets of targets. The targets of each network community were also classified based on protein families, defined in IUPHAR. Multilevel clustering was used as the community detection method. This method is a “greedy” algorithm—that is, it creates an optimal solution at each step to find a good global optimum—that creates communities based on maximizing modularity and is recommended for networks of this size. This method is also appropriate for unconnected networks and can use edge weights to determine community structure (Yang et al., 2016).

### Analysis Tools

The analysis was conducted using R (v3.3.1) (https://www.r-project.org/). Packages used included dplyr (v0.7.4) (https://dplyr.tidyverse.org) and iGraph (v1.1.2) (http://igraph.org). Network visualization was also performed in Cytoscape (v3.4.0) (http://cytoscape.org).

## Results

### Natural Product Data Distributions

Our final set of natural products, compiled from TarNet and TCMID, contained 50,109 compounds, uniquely identified by the five keys found in the source databases: chemical name, SMILES, INCHIKEY, Pubchem Compound ID and CAS number. Of these compounds, target interaction data was retrieved for 4,991 from seven public databases. To contrast this with approved cancer drug space, there are only 137 anti-neoplastic drugs in the Cancer Targetome, which included all FDA approved drugs for cancer as of 2017.

**Figure 2:**
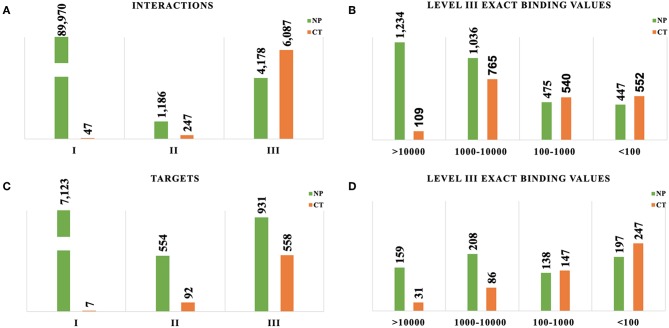
Assessment of evidence levels for target and compound-target interactions. CT, Cancer Targetome; NP, Natural Product Target Network. **(A)** Comparison of interaction distribution by evidence level between NPs and CT drugs. **(B)** Comparison of interaction distribution at high affinity only (evidence level III exact values in nM). **(C)** Comparison of maximum target evidence level distribution between NPs and CT drugs. **(D)** Comparison of maximum target evidence level distribution at high affinity only (evidence level III exact values in nM).

Most of the NP-target interactions were classified as Evidence Level I (94%), and most CT interactions were classified as Evidence Level III (95%), although the absolute counts of interactions were much larger for NPs given the overall number of NPs (Figure 2A). For stronger affinity interactions (Evidence Level III Exact), the raw numbers of interactions were comparable between natural products and cancer drugs (Figure 2B). The large volume of Level I evidence for NPs is primarily from the TarNet database, which is populated via text mining of the public literature for natural products, combined with manual curation. All of these interactions are supported by literature references (and therefore technically are Evidence Level II), but the linkage to these literature references is not made publicly available in the TarNet database, therefore these interactions are restricted to Evidence Level I due to accessibility issues. Curation of a random sampling of these data showed that approximately 37% of the supporting literature has binding data available and would therefore could potentially be assigned Level III were the linkage to this supporting evidence made accessible.

Targets are reported according to the highest evidence level of all of the associated compound interactions for each target (Figure 2C). This approach allows us to take a target-focused perspective and determine the maximum strength of evidence supporting any compound/drugs interacting with it. For a target set of interest, this framework now us to prioritize those targets with stronger evidence for natural product or drug interactions. As with the target-interaction distribution, most of the NP associated targets had a maximum Evidence Level I (83%) and most of the CT drug associated targets had a maximum Evidence Level III (85%), but the distributions were more comparable within Evidence Level III (Figure 2D).

### Natural Product Targets and Cancer Targetome Targets Show Similar Topology Characteristics in Selected Biological Networks

In addition to descriptively evaluating pathway and target coverage by NP compounds compared to coverage by FDA-approved antineoplastic drugs, it is also important to consider the target sets associated with NPs and CT drugs in the context of molecular interaction networks, such as PPI networks. The topology of these networks can be related to biological function (Winterbach et al., 2013). Some topology measures commonly considered relevant to biological function include degree centrality, betweenness centrality, eigenvector centrality, average shortest paths, and clustering coefficients (Zhu et al., 2007; Pavlopoulos et al., 2011). These measures, and others, have been suggested as a guide to identify potential therapeutic targets for drug development (Hwang et al., 2008; Arrell and Terzic, 2010; Winterbach et al., 2013; Peng and Schork, 2014).

Two biological networks (PPIs) were used to evaluate functional relevance of the targets. One is the Reactome Functional Interaction network, and the other was created by Wang (2014) based on interactions specific to cancer biology. The three target node categories include nodes targeted by NPs only, targeted by CT drugs only, or targeted by both CT drugs and NPs. In Reactome, these three node categories represented 393 of the 12,227 total nodes contained in the network. In the wang (cancer) network, these three node categories represented 364 of the 6,306 total nodes in the network. Comparisons were done for the following topology measures: degree, betweenness, and eigenvector centrality.

### Betweenness Centrality

In the Reactome network, betweenness for all three target node categories was significantly higher than it was for non-targeted nodes in the network. There was no significant difference between these three categories. Consistent with previous research, betweenness was significantly higher for the cancer driver nodes than for non-driver nodes in this network.

In the wang (cancer) network (Wang, 2014), as with the Reactome network, all three target node categories had higher betweenness than the non-target nodes. The nodes targeted only by NPs were not different from the nodes targeted only by CT drugs, but the betweenness for these nodes was less than that for the nodes targeted by both NPs and CT drugs. Consistent with previous research, the cancer driver nodes were also significantly higher for betweenness than the non-cancer driver nodes.

The results for this statistic were mostly consistent between the two networks. Overall, nodes targeted by NPs are similar for this characteristic to nodes targeted by CT drugs, and different from non-targeted nodes. The average value for the nodes targeted by both NPs and CT drugs was higher for this measure than the average for nodes targeted by either NP only or CT drugs only, although this difference was not significant when compared to CT only drug targets, and there were only 39 nodes in this category.

Betweenness measures the number of shortest paths in a network that pass through a given node. The target with the highest betweenness in the Reactome network, interacting with only NPs at binding affinity < 100 nM, was nuclear factor kappa beta subunit 1 (NFKB1). The interacting NP compound was rocaglamide, extracted from a variety of *Aglaia* plant species. This gene functions in a dozen large molecular pathways, ranging in size from 72 to 758 genes, most of which are enriched with cancer drivers. This protein is a transcription factor found in almost all cell types and is involved in a wide variety of cell processes, including some that are related to cancer, such as inflammation, tumorigenesis, apoptosis and cell growth. This protein is activated by many intracellular and extracellular stimuli, which is consistent with a high degree of betweenness centrality.

A literature search for the natural product “rocaglamide” returned 68 articles, many of which were *in-vitro* and mouse xenograph cancer studies with positive results. Cancers tested with positive results included renal cell carcinoma (Nalli et al., 2018), non-small cell lung cancer (Yao et al., 2018), multiple myeloma, T-cell leukemia (Wu et al., 2017). This natural product was also found to protect non-malignant cells (Becker et al., 2014). This body of literature also addressed infectious disease applications for this compound.

### Degree Centrality

In the Reactome network, degree was not significantly different between all three target node categories, but each of these three categories were all significantly higher than non-targeted nodes in the network, as was seen with betweenness. The cancer driver nodes were also higher for this measure than non-driver nodes.

In the wang (cancer) network, the nodes targeted by NPs only were not significantly different from non-targeted nodes, and they were significantly lower for this measure than nodes targeted by CT drugs only or nodes targeted by both NPs and CT drugs. The cancer driver nodes were also significantly higher for this measure in this network.

For degree centrality, the two networks were not in agreement. The nodes targeted only by NPs were similar to CT drug nodes in the Reactome network, but not in the wang (cancer) network. But nodes targeted by both NPs and CT drugs were again higher on average than either of the other two categories, NP only and CT drugs only, although it was only significant for the comparison to the NP-only targeted nodes.

The node with the highest degree targeted only by NPs at a binding affinity < 100 nM in the Reactome network is the same as the top node for betweenness for this category, nuclear factor kappa beta subunit 1 (*NFKB1*). In this network, there were 515 interactions with other proteins for this protein target. Of these 515 proteins, 90 (17.5%) were cancer drivers.

### Eigenvector Centrality

For this measure, in the Reactome network, nodes targeted only by NPs were not different from non-targeted nodes, were significantly higher than nodes targeted by CT drugs only, and significantly lower than nodes targeted by both CT drugs and NPs. The nodes targeted only by CT drugs were significantly lower than non-targeted nodes, but not different from nodes targeted by both CT drugs and NPs. The nodes targeted by both CT drugs and NPs were significantly higher than non-targeted nodes. Cancer driver nodes were significantly higher for this measure than non-cancer driver nodes in this network.

In the wang (cancer) network, nodes targeted only by NPs were not different from non-targeted nodes but were significantly less than nodes targeted only by CT drugs and nodes that were targeted by both CT drugs and NPs. Nodes targeted by CT drugs only and those targeted by both CT drugs and NPs were significantly higher than non-targeted nodes. CT only nodes were not different from nodes targeted by both CT drugs and NPs. Cancer driver nodes were significantly higher than non-cancer driver nodes for this measure.

The two networks were not in agreement for these node categories for this measure. Generally, nodes targeted by NP only were not the same as other targeted nodes in both networks, but the direction of these differences was not the same. This was partly because the CT drug only nodes in the Reactome network were generally lower than most other categories, including non-targeted nodes.

The node targeted only by NPs that exhibited the highest eigenvector centrality in the Reactome network was Heterogeneous Nuclear Ribonucleoprotein A1 (*HNRNPA1*). This means that the other nodes directly connected to *HNRNPA1* had a high level of connectivity. There were 185 proteins interacting with this protein in this network, with an average degree of 217, which was significantly higher than the network average of 38. While there were only 27 cancer drivers in this list, or 15% of the 185 proteins, one of these proteins, E1A Binding Protein P300 (*EP300*), has over 1000 interactions listed in Reactome. *HNRNPA1* is an abundant core protein found in the cell nucleus and functions in alternative splicing of RNA.

There was a high level of overlap for the top genes ranked by betweenness and degree centrality for the three target categories (Table 2). There was less overlap between these genes and the genes with the highest eigenvector centrality.

**Table 2:** Top genes for the three topology measures (betweenness, degree, eigenvector centrality).

**NP only**			**CT only**			**Both CT and NP**		
**Gene**	**Value**	**Driver?**	**Gene**	**Value**	**Driver?**	**Gene**	**Value**	**Driver?**
**BETWEENNESS CENTRALITY**								
Nuclear Factor Kappa B Subunit 1 (NFKB1)	901,715	No	SRC Proto-Oncogene, Non-Receptor Tyrosine Kinase (SRC)	911,785	Yes	Epidermal Growth Factor Receptor (EGFR)	947,674	Yes
RELA Proto-Oncogene, NF-KB Subunit (RELA)	613,037	No	FYN Proto-Oncogene, Src Family Tyrosine Kinase (FYN)	616,175	No	Histone Deacetylase 1 (HDAC1)	669,591	No
Cyclin Dependent Kinase 1 (CDK1)	532,453	No	Janus Kinase 2 (JAK2)	504,217	Yes	Estrogen Receptor 1 (ESR1)	575,569	Yes
MDM2 Proto-Oncogene (MDM2)	395,265	Yes	Retinoid X Receptor Alpha (RXRA)	460,453	No	Histone Deacetylase 2 (HDAC2)	510,638	Yes
Protein Kinase C Beta (PRKCB)	315,021	No	Mitogen-Activated Protein Kinase 14 (MAPK14)	389,578	No	Androgen Receptor (AR)	445,269	Yes
Protein Kinase C Alpha (PRKCA)	251,601	No	Retinoic Acid Receptor Alpha (RARA)	300,939	Yes	Cyclin Dependent Kinase 4 (CDK4)	299,008	Yes
**DEGREE CENTRALITY**								
Nuclear Factor Kappa B Subunit 1 (NFKB1)	521	No	SRC Proto-Oncogene, Non-Receptor Tyrosine Kinase (SRC)	569	Yes	Epidermal Growth Factor Receptor (EGFR)	569	Yes
RELA Proto-Oncogene, NF-KB Subunit (RELA)	470	No	FYN Proto-Oncogene, Src Family Tyrosine Kinase (FYN)	497	No	Histone Deacetylase 1 (HDAC1)	497	No
Cyclin Dependent Kinase 1 (CDK1)	468	No	Janus Kinase 2 (JAK2)	398	Yes	Histone Deacetylase 2 (HDAC2)	398	Yes
Protein Kinase C Alpha (PRKCA)	297	No	Mitogen-Activated Protein Kinase 14 (MAPK14)	332	No	Histone Deacetylase 3 (HDAC3)	332	Yes
Protein Kinase C Beta (PRKCB)	296	No	Phosphatidylinositol-4,5-Bisphosphate 3-Kinase Catalytic Subunit Delta (PIK3CD)	324	Yes	Cyclin Dependent Kinase 4 (CDK4)	324	Yes
MDM2 Proto-Oncogene (MDM2)	260	Yes	LYN Proto-Oncogene, Src Family Tyrosine Kinase (LYN)	311	No	Estrogen Receptor 1 (ESR1)	311	Yes
**EIGENVECTOR CENTRALITY**								
Heterogeneous Nuclear Ribonucleoprotein A1 (HNRNPA1)	0.77	No	Retinoic Acid Receptor Alpha (RARA)	0.14	Yes	Histone Deacetylase 3 (HDAC3)	0.13	Yes
Cyclin Dependent Kinase 7 (CDK7)	0.25	No	Aurora Kinase B (AURKB)	0.13	No	Histone Deacetylase 1 (HDAC1)	0.10	No
Cyclin T1 (CCNT1)	0.18	No	Retinoid X Receptor Alpha (RXRA)	0.12	No	Epidermal Growth Factor Receptor (EGFR)	0.09	Yes
Cyclin Dependent Kinase 9 (CDK9)	0.17	No	Nuclear Receptor Corepressor 1 (NCOR1)	0.12	Yes	Histone Deacetylase 2 (HDAC2)	0.08	Yes
Cyclin Dependent Kinase 1 (CDK1)	0.17	No	SRC Proto-Oncogene, Non-Receptor Tyrosine Kinase (SRC)	0.10	Yes	Cyclin Dependent Kinase 4 (CDK4)	0.07	Yes
RELA Proto-Oncogene, NF-KB Subunit (RELA)	0.11	No	Retinoic Acid Receptor Beta (RARB)	0.09	No	Hypoxia Inducible Factor 1 Alpha Subunit (HIF1A)	0.06	No

*The top 6 genes with the highest values of betweenness, degree and eigenvector centrality are shown for each node category: targeted by NP only, targeted by CT drugs only, or targeted by both NP and CT drug. All interactions are < 100 nM binding affinity*.

In both networks, the average shortest path distances from either NP only, CT drug only, and both CT/NP nodes to cancer driver nodes were significantly shorter than a set of randomly selected nodes. The nodes targeted by both CT drugs and NPs were the closest to the cancer driver nodes, on average. This proximity to cancer drivers indicates fewer reaction steps between the targeted node and the cancer driver node and greater potential to interfere with an oncogenic signal from the driver. These nodes also had higher betweenness and degree. These nodes represent a subset of both CT drug nodes and NP nodes that appear to be more critical in both networks. These 39 nodes also contained a higher percentage of cancer drivers than the other categories.

### Pathway Interactions Reveal Potential Synergistic Relationships Between Natural Products and Cancer Drugs

The use of compensatory and redundant molecular pathways by the cancer cell is a likely mechanism of acquired drug resistance, suggesting that rational drug combination therapy could inhibit these processes. Several methods have been proposed to select and predict drug combinations that could inhibit this phenomenon (Peng and Schork, 2014; Sun et al., 2015; Chen et al., 2016; Jaeger et al., 2017).

For this analysis, we used the pathway network model developed by Chen et al. using shared genes as the criterion for an edge between two pathway nodes. Our network contained 285 pathways (nodes) with 9,152 edges. These 285 were non-hierarchical Reactome entity-level pathways. Of the 285 pathways, 90 were enriched with cancer drivers. The majority of the cancer pathways were targeted by both NPs and CT drugs (73%), and most of the non-cancer pathways were not targeted by either NPs or CT drugs (48%). Only target interactions < 100 nM were considered.

The average shortest path in this network between pathways targeted by CT drugs, those targeted by NPs, and between NP-targeted pathways and CT-drug targeted pathways were all similar, and closer than random controls. Based on previous research, this short distance could correlate with synergy between these classes of compounds, particularly between NPs and CT drugs. Pathways targeted by both CT drugs and NPs were closer on average to cancer pathways than random controls. Pathways targeted by NPs only and those targeted by CT drugs only were not closer to cancer pathways than random controls (Supplemental S1).

There were 559 examples of neighboring pathways (nodes) in this network, where one pathway is targeted by NPs only and the other pathway is a cancer-enriched pathway targeted by either CT drugs or both CT drugs and NPs. An example is **DAG and IP3 signaling** and **Signaling by FGFR1**. In the Reactome database, these two pathways share the gene PLCG1 (1-phosphatidylinositol 4,5-bisphosphate phosphodiesterase gamma-1), which created an edge between them in this network. The pathway **Signaling by FGFR1** was enriched with cancer drivers and had a CT drug target with an interaction < 100 nM, BRAF (B-Raf Proto-Oncogene, Serine/Threonine Kinase). This pathway initiates intracellular signaling pathways involved with cell proliferation and migration, and other functions. **DAG and IP3 signaling** was not enriched with cancer drivers and did not have any low affinity CT drug targets, but did have six high affinity NP targets: *PRKCG, E,A,D* (Protein Kinase C), *PDE1A* (Phosphodiesterase 1A) and *ADCY1* (Adenylate Cyclase 1). *DAG* (diacylglycerol) and *IP3* (inositol 1,4,5-trisphosphate) are secondary messengers used in intracellular signaling.

### Natural Products Show Target Family Groupings Both Distinct From and in Common With Cancer Drugs

There was a total of 253 compounds and 1,238 edges in the compound network, which included 68 CT drugs and 185 NPs. This network contained 26 unconnected subnetworks, with the majority of nodes (163) in one large connected component. Multilevel clustering created 35 communities, most of which contained 5 or fewer compounds. Of interest were the three largest communities, each containing over 30 compounds. **One** community was dominated by CT drugs, but also contained a substantial number of NPs. The other two communities contained primarily NPs.

The first community contained 32 CT drugs and 11 NPs. The community clustered primarily around kinase and other cancer-related target families (Table 3). Kinases are extremely well-targeted by current FDA-approved cancer drugs and have been an active area of research following the breakthrough kinase inhibitor imatinib. The next largest community comprised 39 NPs and 4 CT drugs. This community contained a variety of cytochrome P450 families and other target families considered therapeutic targets, such as the carbonate dehydratases, which are inhibited for the treatment of glaucoma and other conditions (Table 3). The third largest community contained no CT drugs and 30 NPs. The target protein families here were primarily hormone and neurotransmitter receptors (Table 3).

**Table 3:** Natural products (NP) and approved cancer drugs (CT) interact with disjoint and shared target sets.

**Largest community (32 CT drugs, 11 NPs)**	**# Targets**	**Second largest community (4 CT drugs, 39 NPs)**	**# Targets**	**Third largest community (0 CT drugs, 30 NPs)**	**# Targets**
Type XIII RTKs: Ephrin receptor family	12	4.2.1.1 Carbonate dehydratases	3	5-Hydroxytryptamine receptors	11
Src family	11	CYP1 family	3	Adrenoceptors	6
Tec family	5	1.-.-.- Oxidoreductases	1	Ionotropic glutamate receptors	4
Type III RTKs: PDGFR, CSFR, Kit, FLT3 receptor family	5	1.13.11.- Dioxygenases	1	Melatonin receptors	2
Death-associated kinase (DAPK) family	4	ABCC subfamily	1	Acetylcholine receptors (muscarinic)	1
HIPK subfamily	4	Aryl hydrocarbon receptor complex	1	Adenosine receptors	1
Janus kinase (JakA) family	4	Carrier proteins	1	CYP2 family	1
KHS subfamily	4	CFTR	1	Dopamine receptors	1
Myosin Light Chain Kinase (MLCK) family	4	Cyclooxygenase	1	Glucagon receptor family	1
RSK subfamily	4	CYP11, CYP17, CYP19, CYP20 and CYP21 families	1	Glutamate transporter subfamily	1
Type I RTKs: ErbB (epidermal growth factor) receptor family	4	Nucleoside synthesis and metabolism	1	Metabotropic glutamate receptors	1

*The top 11 target families in the three largest compound-compound network communities are shown, along with the NP/CT distribution in each. The largest community is dominated by approved cancer drugs and the other two communities are dominated by NPs*.

### Natural Products Increase Coverage of Cancer Pathways, Targets, and Tumor Types

Coverage is assessed at two thresholds: (1) at all levels of supporting evidence, and (2) for when restricting to interactions supporting by binding evidence < 100 nM. Of the 1,944 hierarchically structured Reactome pathways, 533 were considered pan-cancer aberrational based on over-representation analysis. For all Reactome pathways, at all levels of evidence, natural products increased coverage by 61%, relative to pathways covered by CT drugs. Reactome pathway coverage at the 100 nM level was increased by 29%, relatively (Table 4). For the aberrational cancer pathways, the NP relative coverage increase for all evidence levels was 12%, and the increase at the 100 nM level was 6% (Table 5). The percentage of aberrational cancer pathways targeted by both NPs and CT drugs was higher than it was for all Reactome pathways, which would be expected since the Cancer Targetome is specific to the disease domain associated with this subset of pathways.

**Table 4:** Consideration of natural product targets increases both pathway and target coverage in all Reactome pathways.

**Evidence and binding**	**# Unique targets**			**# Reactome pathways targeted**			**% of Reactome pathways**		
	**NP only**	**CT only**	**Both**	**NP only**	**CT only**	**Both**	**NP only (%)**	**CT only (%)**	**Both (%)**
Evidence levels I, II, III	7,978	27	630	725	0	1196	37.29	0.00	61.52
Evidence levels II, III	964	129	521	385	21	1174	19.80	1.08	60.39
Evidence levels III	453	80	478	275	30	1070	14.15	1.54	55.04
Evidence level III, binding LT100	160	210	37	218	266	495	11.21	13.68	25.46

*This pathway data includes hierarchically nested Reactome pathways*.

**Table 5:** Consideration of natural product targets increases both pathway and target coverage in cancer pathways.

**Evidence and binding**	**# Targets in cancer pathways**			**# Cancer pathways**			**% of cancer pathways**		
	**NP only**	**CT only**	**Both**	**NP only**	**CT only**	**Both**	**NP only (%)**	**CT only (%)**	**Both (%)**
Evidence levels I, II, III	3,488	16	387	59	0	474	11.07	0.00	88.93
Evidence levels II, III	422	87	313	24	2	472	4.50	0.38	88.56
Evidence levels III	250	51	279	35	4	450	6.57	0.75	84.43
Evidence level III, binding LT100	102	145	26	24	97	286	4.50	18.20	53.66

*The cancer pathways include 533 pathways and 7,339 associated targets from the Reactome hierarchically nested pathways*.

**Figure 3:**
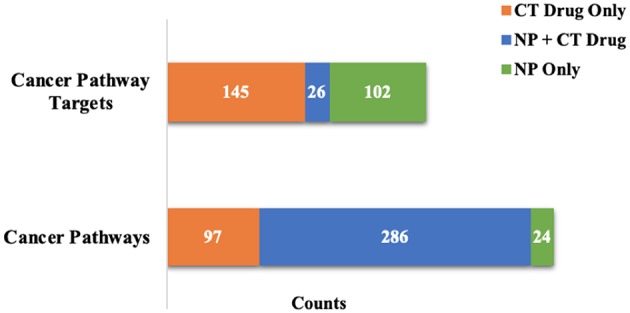
Evaluation of cancer pathway overlap at affinities <100 nM. There is a high level of pathway overlap between those targeted by NPs and CT drugs, but little overlap at the individual target level.

When considering the NP contributions within pathways targeted by both NPs and CT drugs, 51% of the individual target interactions in the 1,196 Reactome pathways were with NPs only. For the 495 Reactome pathways targeted by both at the 100 nM level, 43% of the target interactions were with NPs only. For cancer pathways targeted with affinities < 100 nM there was a high degree of overlap by compound classes at the pathway level, but very little overlap at the target level (Figure 3). Not only were the number of pathways targeted increased when considering the NP target space, but coverage of targets within pathways was increased in those pathways already targeted by CT drugs.

When considering all of the targets contained in both target networks, a large number of interactions were with NPs only (Table 4). We found the same result for the 7,339 targets associated with the aberrational cancer pathways (Table 5). The vast majority of these interactions were Level I evidence from the TarNet database. At the stronger binding affinity (< 100 nM), there is a relative increase in target coverage of 65% for all targets from the two networks, and 60% for targets from the aberrational cancer pathways.

Target interactions were then assessed for the 837 cancer drivers, which are then mapped back to their associated tumor types. There were twelve tumor types for which NPs increased driver coverage (Table 6). For these tumor types, five cancer drivers were targeted only by NPs at < 100 nM, including Adenylate Cyclase 1 (*ADCY1*), Matrix Metallopeptidase 2 (*MMP2*), Aryl Hydrocarbon Receptor (*AHR*), Cyclin Dependent Kinase 2 (*CDK2*), and Mitogen-Activated Protein Kinase 11 (*MAP3K11*). Some of the plant derived NPs targeting these drivers include forskolin (from the Indian coleus plant, *ADCY1*), caffeic acid (found in many foods including coffee, *MMP2*), kaempferol (found in many common foods including apples, grapes, tomatoes and green tea, AHR), and flavopiridol (a semi-synthetic derivative from the Pithraj tree, *CDK2*). *MAP3K11* is targeted by staurosporinone, which is of bacterial origin so is out of the scope of the NP definition for this paper. While there is a significant body of *in-vitro* cancer research associated with these natural products, there are 65 clinical trials listed for flavopiridol, many of which are either single agent or combination trials for cancer^2^.

**Table 6:** Natural products improve coverage of cancer drivers across cancer types.

**Tumor type**	**CT only**	**NP only**	**Both NP and CT**	**Total cancer drivers**
Cutaneous melanoma	20	2	4	291
Prostate adenocarcinoma	10	2	3	153
Bladder	10	1	2	195
Esophagous	3	1	3	124
Head and neck squamous	9	1	4	188
Hodgkin lymphoma	0	1	0	12
Large B-cell lymphoma	1	1	0	3
Lung adenocarcinoma	18	1	3	209
Neuroblastoma	2	1	0	31
Renal clear cell	6	1	2	116
Small cell lung	2	1	0	59
Uterine corpus endometroid carcinoma	9	1	4	158

*Twelve tumor types have increased driver coverage from NPs, at binding values of 100 nM or less. The ‘NP Only' column lists the number of drivers targeted only by NPs*.

**Figure 4:**
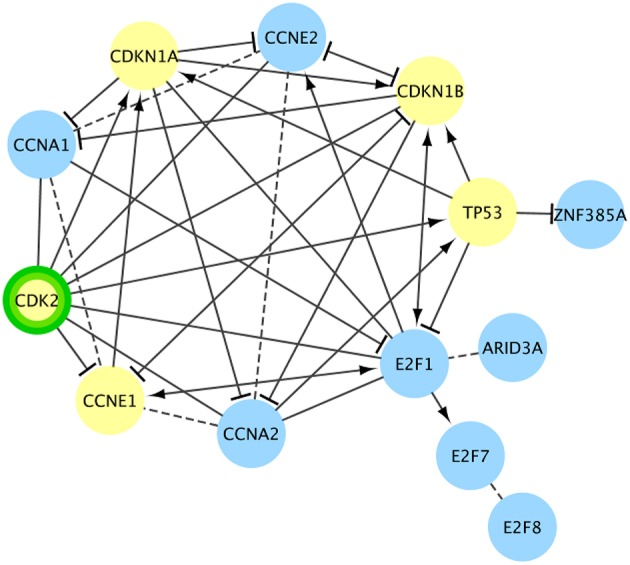
Cancer pathway gene set targeted only by natural products for a cancer driver target. Another Reactome functional interaction network (**TP53 Regulates Transcription of Genes Involved in G1 Cell Cycle Arrest**) targeted only by NPs at <100 nM (green border). No FDA-approved cancer drugs target this pathway with < 100 nM evidence. Cancer drivers are shown in yellow. In this pathway the NP target is also a cancer driver. Dashed lines are predicted interactions.

There were 24 pathways in Reactome that were enriched for cancer drivers, had no CT drug interactions at < 100 nM, and had at least one target interaction with a NP with a binding affinity < 100 nM. In some of these pathways, such as **“RUNX1 regulates transcription of genes involved in differentiation of myeloid cells**,” the NP interaction was not with a driver gene (Supplemental S2). In this example, the interacting NP is a phorbol ester isolated from croton oil. Phorbol esters have been called “double-edged swords,” showing both tumor promoting and tumor inhibiting activities, depending on the cancer (Goel et al., 2007). This pathway is involved with the differentiation of myeloid progenitors and also with apoptosis of mature myeloid cells (Ntie-Kang et al., 2013). The three driver genes in this pathway are associated with over 20 tumor types, including acute myeloid leukemia, acute lymphoblastic leukemia, chronic myeloid leukemia, myelodysplastic syndrome, and non-Hodgkin's lymphoma. In other pathways, such as **“TP53 Regulates Transcription of Genes Involved in G1 Cell Cycle Arrest**,” the NP interaction was directly with a driver gene (Figure 4). The interacting NP for this example is flavopiridol, which is a semisynthetic derivative of a compound extracted from the Pithraj tree. This compound is already in numerous clinical trials for several cancer types (Tse et al., 2016). This pathway primarily inhibits the cell cycle transition from the G1 phase to the S phase through multiple mechanisms (Ntie-Kang et al., 2013). Over 40 tumor types are associated with the five cancer drivers in this pathway, including breast, bladder, esophagus, head and neck, lung, prostate, ovary, and hepatocellular.

There were also 286 pathways in Reactome that were enriched for cancer drivers that were targeted by both NPs and CT drugs at binding affinities < 100 nM. One smaller pathway example of this is **“MAPK3 (ERK1) activation”** (Supplemental S3). The two NP compounds targeting the three proteins in this pathway are flavopiridol, and arctigenin, which is extracted from the burdock plant. This pathway is involved in a wide range of cellular processes, including cytoskeleton remodeling, proliferation, differentiation and regulation of inflammatory responses (Ntie-Kang et al., 2013). Over 20 tumor types are associated with the four cancer drivers found in this pathway, including leukemias, lung cancers, colorectal cancer, melanoma, and head and neck squamous cell cancer.

We found significant differences between the distributions of the number of targets and pathways interacting per compound for NPs vs. CT drugs. Additionally, we found that the significant distribution differences for tumor types per compound and cancer driver interactions per compound were dependent on the binding affinity threshold (Table 7). But it is difficult to draw any further conclusions about these differences, since the motivations for studying the relationships are not necessarily the same between NPs and FDA-approved cancer drugs. Different experiments could be designed to compare promiscuity by running matched assay panels on both NPs and FDA-approved drugs. Previous research has shown an increasing number of target interactions per compound as they progress along the drug development pipeline, with approved drugs having the highest number of interactions (Hu and Bajorath, 2013; Hu et al., 2014). Importantly, we note that the total number of targets tested for each of these compounds (for both NPs and CT drugs), is not captured in the public data resources used for this project. This information is important both to explain therapeutic polypharmacological effects and undesirable adverse events. Finally, there is always the problem of incomplete data for this characteristic since all possible target interactions that occur in a biological context cannot be known.

**Table 7:** Distribution comparisons for targets, pathways, tumors and cancer drivers.

**Type**	**Affinity level**	**KS test**	
		***p***	***D***
Targets	LT100 all targets^*^	0.002222	0.26364
	LT1000 all targets^*^	0.0002767	0.27611
	LT100 cancer pathway targets^*^	0.003176	0.28632
	LT1000 cancer pathway targets^*^	0.001013	0.27778
Pathways	LT100 all pathways^*^	7.37E-12	0.52679
	LT1000 all pathways^*^	3.28E-13	0.51282
	LT100 cancer pathways^*^	2.35E-10	0.54144
	LT1000 cancer pathways^*^	9.88E-13	0.54069
Tumors	LT100	0.02306	0.45714
	LT1000^*^	0.0008806	0.42083
Drivers	LT100	0.1024	0.37302
	LT1000^*^	0.003771	0.37917

*Differences between the number of targets, pathways, cancer drivers and tumors interactions per compound for NPs and CT drugs were tested using a two sample Kolmogorov Smirnov test to detect differences in the distributions. This testing was assessed for binding levels of 100 nM or less, and 1000 nM or less. Tumor associations made via cancer driver interactions*.

* *indicates significance at p threshold of < 0.004 (Bonferroni adjustment)*.

## Discussion

Natural products show much potential for combination therapy in cancer treatment; but barriers in the public domain of natural product-target interactions make it difficult to harness this information in a high throughput manner. Here, we applied an evidence framework to public domain natural product information and quantitatively assessed the added value of natural product space when used in conjunction with approved cancer drug space. Natural products increase the cancer-relevant targetable space from both a target and a pathway perspective, as well as providing additional coverage of key cancer driver genes. Given current challenges in acquired drug resistance to approved cancer drugs, the inclusion of natural products in combination therapies may allow us to broaden our scope of targetable cancer-relevant pathways for therapeutic advantage.

We have demonstrated that the projection of this natural product target network into various biological contexts can identify candidate natural products for cancer treatment, and provided examples with substantial support in the literature. This previous research is most likely the source of some of the public target data we used. To test the ability of this network to identify novel natural product cancer candidate treatments that have not been previously reported, we are projecting these network targets into molecular pathways associated with head and neck oral squamous cell cancer (HNSCC), identified by using the HNSCC cohort data from The Cancer Genome Atlas. Further, we are testing cell viability of patient derived HNSCC tumor cells exposed to over 160 cancer drugs and natural products. In preliminary analyses we identified two natural products among the top five compounds to which one patient's tumor cells were sensitive, the other three being cancer drugs approved or under development (Vigoda, unpublished). These findings are encouraging for the potential of these public data, efficiently mined through the methods detailed herein, to reveal new natural product candidates for cancer treatment.

The natural product-target interaction space in the public domain still faces challenges that will need addressing in order to more fully harness the therapeutic potential of natural products. For instance, our work here brought attention to several key quality issues in publicly available resources for natural product–target interaction data. The two databases chosen, TarNet and TCMID, are not the only available for NPs, but they were appropriate choices given our criteria for defining natural products. The heavy reliance on text mining for these resources could explain some of the data quality issues that we encountered. For instance, some of these data sources appear to contain drugs that do not have a “true” NP source, or include compounds used in the extraction of these NP compounds. For this reason, we removed any compounds with a synonym match to drugs using FDA and DrugBank compound lists. We also manually curated our high binding affinity natural product compounds and removed about 11% due to quality concerns, such as the inclusion of synthetic compounds. Another issue encountered with these data sources was the inability to download the entire database from the website. There are well-curated proprietary natural product databases, such as NapAlert and The Dictionary of Natural Products, for future application of these methods and findings beyond our current analyses including only public resources.

While we did find an increase in relevant target and pathway coverage by considering NP target interactions in public data sources, it is also important to consider and compare these two target spaces (CT and NP) in a biological context. We used two networks to do this, the Reactome network and the cancer-specific network. Of these two, the Reactome network is more comprehensive. In this network, the new targets identified by considering NPs were similar to drug-targeted nodes and less similar to non-targeted nodes, when considering betweenness and degree. This similarity was not seen for eigenvector centrality. Degree and betweenness are strong measures of criticality in a network and the fact that the new NP targets are similar to CT drug targets could indicate potential for novel combination therapies. Also, of interest is that the nodes targeted by both NPs and CT drugs tended to have higher average values for these measures than either CT drug only or NP only targets.

In general, biological networks are scale-free, meaning that many nodes have low connectivity and are not critical to the function of the network, but some nodes are highly connected and more critical, possibly making good therapeutic targets. Topology measures can guide the identification of these critical nodes. Both degree and betweenness are considered measures of strong importance to the network, but nodes with high values of either can be considered too lethal or toxic to target (Hwang et al., 2008; Arrell and Terzic, 2010; Winterbach et al., 2013; Peng and Schork, 2014). It is possible to identify nodes with network influence that have lower essentiality (Winterbach et al., 2013) by using measures such as eigenvector centrality, bridging centrality, and others (Hwang et al., 2008; Arrell and Terzic, 2010; Peng and Schork, 2014). Eigenvector centrality is a measure of the connectivity of the nodes that are connected to the node of interest. Bridging centrality, developed by Hwang et al. is a measure of how well a node connects separate modular sub-regions in a network. Other strategies for reducing adverse effects consider targets that influence critical nodes without targeting the critical nodes themselves (Peng and Schork, 2014; Laderas et al., 2015). For this reason, assessing the shortest path distances from critical nodes, such as cancer driver genes, is important. This distance could also be considered an estimate of the number of molecular steps from this node (Yildirim et al., 2007). It has also been shown that cancer driver genes have topology measures in biological networks that are distinctly different from other nodes. These differences include higher degree and betweenness, shorter paths between them and weaker clustering coefficients (Sun and Zhao, 2010; Xia et al., 2011; Winterbach et al., 2013).

Biological network topology has also been used to predict therapeutic synergy between compounds, an aspect of interest for designing novel combination therapies (Yin et al., 2014). Several *in-silico* methods for prediction of synergy in combination therapy use topology measures (Li et al., 2011; Huang et al., 2014; Sun et al., 2015). Generally, higher values of measures, such as degree and betweenness, are preferred in these models.

The clustering patterns seen in the larger communities in the compound-compound network seem to indicate NP target interaction research driven by known therapeutic targets as seen in drug therapeutic classifications. Since the only therapeutic classification for the drugs used in this study is cancer, it follows that there is one large cluster containing a majority of the CT drugs and also some NPs, indicating interest in NPs for cancer research. If this analysis were expanded to include drugs from other therapeutic classifications, the other communities might also contain the associated drugs, along with the NPs. The drugs from these other therapeutic classifications could also be investigated for cancer therapies. In this study, we did not investigate non-cancer drugs that interact with the targets found to interact only with NPs. That we saw compound clustering around target families common to NPs and CT drugs, and also clustering around families unique to NPs, when compared to CT drugs, supports the potential of complementary therapeutic relationships between the two compound classifications.

From the perspective of combination therapy and synergy discovery, the focus of this research was on a pharmacodynamic framework. There is a large body of literature for predictive algorithms for combination therapies that is based on network pharmacology, which influenced this work. Many of these methods use compound-target networks, such as what we have developed for natural products, to derive the predictive features used in these models. However, NPs can also interact with compounds, either synergistically, additively, or antagonistically, through other mechanisms. These can include pharmacokinetic interactions, drug efflux transporter interactions, and other cell microenvironment interactions. Furthermore, some synergy effects of NPs are not mediated by direct interaction with a protein target, but through the up- or down-regulation of the expression of the protein of interest. Since binding assay results do not necessarily provide functional information about the compound-target interaction, the presence of a strong binding assay may not necessarily indicate a positive therapeutic effect for cancer. In fact, some of the interactions could promote tumor growth, as is the case in some cancers whose growth can be stimulated with phorbol esters isolated from croton oil (Goel et al., 2007). Future efforts should consider both the strength of compound-target binding and the functional effect of the interaction. All of these mechanisms could be considered in future algorithms to predict combination therapies.

Overall, the intent of this work was to assess both the natural product therapeutically targetable space and also to assess the potential complementarity of natural products with FDA-approved cancer drugs. As discussed previously, while we were often not able to verify the direct link between supporting literature and the large amount of Level I interactions due to accessibility issues, our random sampling of this space indicates that a sizeable portion of this Level I evidence is actually supported by literature and/or binding assay values. This presents a high need and actionable area for natural product-target curation. Improved curation and accessibility of natural product-target interactions will greatly enable efforts to harness natural products in the cancer therapeutic space. While further work is needed to improve the quality of evidence associated with natural product target interactions, our work here shows that there is a clear benefit to the inclusion of natural products along with FDA-approved cancer drugs in this effort.

## Author Contributions

SC, AB, and SM conceived of the presented idea. SC and AB developed the framework. SC, AB, and GC conducted the analyses. SC, AB, GW, LS, GC, MK-M, and SM contributed to the study design, development of analysis plan, as well as development and refinement of the manuscript.

### Conflict of Interest Statement

The authors declare that the research was conducted in the absence of any commercial or financial relationships that could be construed as a potential conflict of interest.

## Footnotes

^1^National Cancer Institute. *Types of Treatment*. Available online at: https://www.cancer.gov/about-cancer/treatment/types

^2^*ClinicalTrials.gov*. Available online at: https://clinicaltrials.gov
