# Supplementary material for: Natural Product Target Network Reveals Potential for Cancer Combination Therapies
Source: Front Pharmacol. 2019 May 31;10:557. doi: 10.3389/fphar.2019.00557 (PMC6555193; doi:10.3389/fphar.2019.00557)
Supplement: Supplementary file 1 [file Data_Sheet_1.docx]

**Data Availability Statement**

1. **Datasets are in a publicly accessible repository:**

The data from DrugBank (Law et al. 2014) used for this study can be found at:

<https://www.drugbank.ca/releases/latest#full>

<https://www.drugbank.ca/releases/latest#external-links>

The data from Therapeutic Targets Database (Qin et al. 2014) used for this study can be found at:

<https://db.idrblab.org/ttd/full-data-download>

The data from the International Union of Basic and Clinical Pharmacology (Harding et al. 2018) used for this study can be found at:

<http://www.guidetopharmacology.org/download.jsp#data>

The data from TarNet (Hu et al. 2016)used in this study can be found at:

<https://figshare.com/s/7b5e1d93f2b6cca89e8e>.

The data from the Traditional Chinese Medicine Integrated Database (TCMID) (Huang et al. 2018) can be found at:

“http://www.megabionet.org/tcmid/”

1. **Restrictions apply to the datasets:**

The following datasets we used are no longer available publicly or we have been asked by the developer not to give out copies. For this reason we would ask that if there is interest in these sources that the developers be contacted directly for permission.

**TarNet (Hu et al. 2016) :** We requested and received additional non-public data from the developer of this dataset. Contact information can be found in the supporting publication:

<https://www.ncbi.nlm.nih.gov/pmc/articles/PMC4919029/>

**Universal Natural Products Database (Gu et al. 2013)** : This database appears to no longer be supported online, although we have downloaded a copy. Contact information can be found in the supporting documentation:

<https://www.ncbi.nlm.nih.gov/pubmed/23638153>

Gu, J., Y. Gui, L. Chen, G. Yuan, H. Z. Lu, and X. Xu. 2013. 'Use of natural products as chemical library for drug discovery and network pharmacology', *PLoS One*, 8: e62839.

Harding, S. D., J. L. Sharman, E. Faccenda, C. Southan, A. J. Pawson, S. Ireland, A. J. G. Gray, L. Bruce, S. P. H. Alexander, S. Anderton, C. Bryant, A. P. Davenport, C. Doerig, D. Fabbro, F. Levi-Schaffer, M. Spedding, and J. A. Davies. 2018. 'The IUPHAR/BPS Guide to PHARMACOLOGY in 2018: updates and expansion to encompass the new guide to IMMUNOPHARMACOLOGY', *Nucleic Acids Res*, 46: D1091-d106.

Hu, R., G. Ren, G. Sun, and X. Sun. 2016. 'TarNet: An Evidence-Based Database for Natural Medicine Research', *PLoS One*, 11: e0157222.

Huang, L., D. Xie, Y. Yu, H. Liu, Y. Shi, T. Shi, and C. Wen. 2018. 'TCMID 2.0: a comprehensive resource for TCM', *Nucleic Acids Res*, 46: D1117-d20.

Law, V., C. Knox, Y. Djoumbou, T. Jewison, A. C. Guo, Y. Liu, A. Maciejewski, D. Arndt, M. Wilson, V. Neveu, A. Tang, G. Gabriel, C. Ly, S. Adamjee, Z. T. Dame, B. Han, Y. Zhou, and D. S. Wishart. 2014. 'DrugBank 4.0: shedding new light on drug metabolism', *Nucleic Acids Res*, 42: D1091-7.

Qin, C., C. Zhang, F. Zhu, F. Xu, S. Y. Chen, P. Zhang, Y. H. Li, S. Y. Yang, Y. Q. Wei, L. Tao, and Y. Z. Chen. 2014. 'Therapeutic target database update 2014: a resource for targeted therapeutics', *Nucleic Acids Res*, 42: D1118-23.
