# Supplementary material for: Natural Product Target Network Reveals Potential for Cancer Combination Therapies
Source: Front Pharmacol. 2019 May 31;10:557. doi: 10.3389/fphar.2019.00557 (PMC6555193; doi:10.3389/fphar.2019.00557)
Supplement: Supplementary file 2 [file Image_1.pdf]

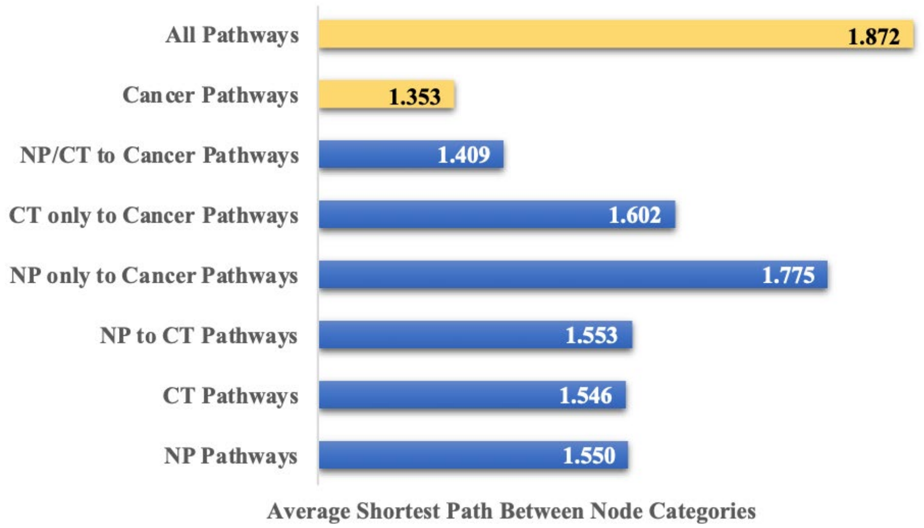

S1. **Pathway-Pathway network analysis.** Average shortest path distances in blue were all shorter than random controls, except for NP only and CT only nodes to cancer nodes (empirical $p<.01$, 1000 permutations).
