# Supplementary material for: Natural Product Target Network Reveals Potential for Cancer Combination Therapies
Source: Front Pharmacol. 2019 May 31;10:557. doi: 10.3389/fphar.2019.00557 (PMC6555193; doi:10.3389/fphar.2019.00557)
Supplement: Supplementary file 3 [file Image_2.pdf]

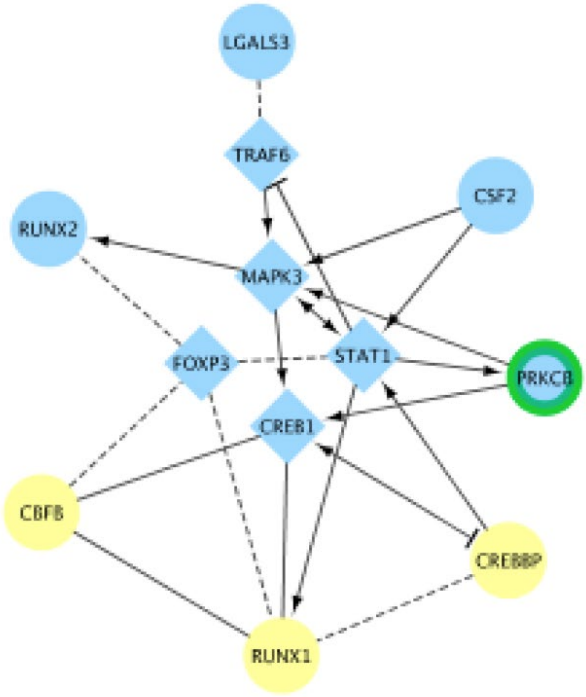

S2. Cancer pathway gene set targeted only by natural products for a non cancer driver target. The Reactome Functional Interaction Network for the **RUNX1 regulates transcription of genes involved in differentiation of myeloid cells** pathway is an example of an aberrant cancer gene set targeted only by NPs at less than 100 nM (green border). No FDA-approved cancer drugs target this pathway with <100nM evidence. Cancer drivers are shown in yellow. The NP target is not a cancer driver. Dashed lines are predicted interactions. The diamond shaped genes are connectors, not part of the formal pathway.
