# Supplementary material for: Natural Product Target Network Reveals Potential for Cancer Combination Therapies
Source: Front Pharmacol. 2019 May 31;10:557. doi: 10.3389/fphar.2019.00557 (PMC6555193; doi:10.3389/fphar.2019.00557)
Supplement: Supplementary file 4 [file Image_3.pdf]

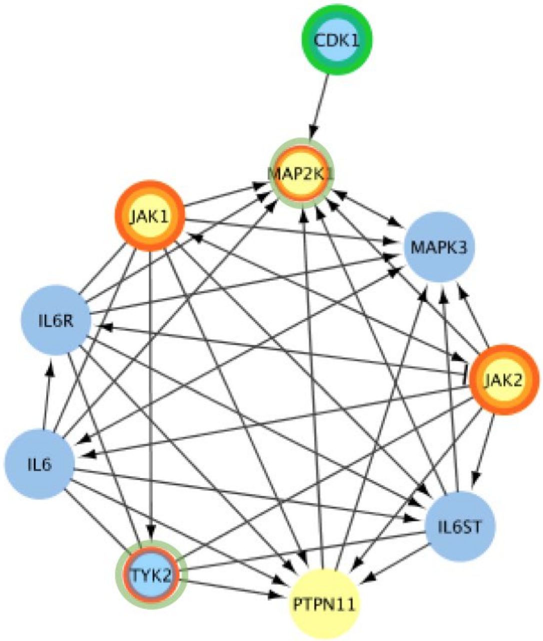

S3. Cancer pathway targeted by both NPs and CT drugs (Wu et al. 2014) . This Reactome pathway (**MAPK3 (ERK1) activation**) is an example of an aberrant cancer pathway targeted by both NPs (3, green borders) and FDA-approved cancer drugs (4, orange borders) at <100nM evidence. Cancer drivers are shown in yellow, non drivers are shown in blue. A border with both green and orange indicates node is a target for both a NP and a CT drug.

Wu, G., E. Dawson, A. Duong, R. Haw, and L. Stein. 2014. 'ReactomeFIViz: a Cytoscape app for pathway and network-based data analysis', *F1000Res*, 3: 146.
